# Identification of an intraocular microbiota

**DOI:** 10.1038/s41421-021-00245-6

**Published:** 2021-03-09

**Authors:** Yuhua Deng, Xiaofei Ge, Yan Li, Bin Zou, Xiaofeng Wen, Weirong Chen, Lin Lu, Meifen Zhang, Xiaomin Zhang, Chunmei Li, Chan Zhao, Xiaofeng Lin, Xiulan Zhang, Xinhua Huang, Xiaorong Li, Ming Jin, Guang-Hua Peng, Dongni Wang, Xun Wang, Weiyi Lai, Juanran Liang, Jing Jing Li, Qiaoxing Liang, Liu Yang, Qinfen Zhang, Yinyin Li, Ping Lu, Xiao Hu, Xifang Li, Xiuli Deng, Yu Liu, Yanli Zou, Shixin Guo, Tingting Chen, Yali Qin, Fuhua Yang, Li Miao, Wei Chen, Chi-Chao Chan, Haotian Lin, Yizhi Liu, Richard W. J. Lee, Lai Wei

**Affiliations:** 1 State Key Laboratory of Ophthalmology, Zhongshan Ophthalmic Center, Sun Yat-sen University, Guangzhou, Guangdong 510060 China; 2 Department of Ophthalmology, Peking Union Medical College Hospital, Chinese Academy of Medical Sciences, Beijing, 100730 China; 3 Tianjin Medical University Eye Hospital, Eye Institute & School of Optometry and Ophthalmology, Tianjin, 300384 China; 4 Department of Ophthalmology, China-Japan Friendship Hospital, Beijing, 100029 China; 5 Department of Pathophysiology, Basic Medical College of Zhengzhou University, Zhengzhou, He’nan 450001 China; 6 Department of Ophthalmology, General Hospital of Chinese People’s Liberation Army, Beijing, 100853 China; 7 State Key Laboratory of Biocontrol, MOE Key Laboratory of Aquatic Product Safety, Institute of Aquatic Economic Animals and Guangdong Province Key Laboratory for Aquatic Economic Animals, School of Life Sciences, Sun Yat-sen University, Guangzhou, Guangdong 510275 China; 8 Department of Biostatistics, University of Pittsburgh, Pittsburgh, PA 15261 USA; 9 Division of Pulmonary Medicine, Allergy and Immunology, Department of Pediatrics, Children’s Hospital of Pittsburgh of UPMC, Pittsburgh, PA 15224 USA; 10 Translational Health Sciences, University of Bristol, Bristol, UK; 11 National Institute for Health Research Biomedical Research Centre at Moorfields Eye Hospital NHS Foundation Trust and UCL Institute of Ophthalmology, London, UK

**Keywords:** Innate immunity, Genomic analysis

## Abstract

The current dogma in ophthalmology and vision research presumes the intraocular environment to be sterile. However, recent evidence of intestinal bacterial translocation into the bloodstream and many other internal organs including the eyes, found in healthy and diseased animal models, suggests that the intraocular cavity may also be inhabited by a microbial community. Here, we tested intraocular samples from over 1000 human eyes. Using quantitative PCR, negative staining transmission electron microscopy, direct culture, and high-throughput sequencing technologies, we demonstrated the presence of intraocular bacteria. The possibility that the microbiome from these low-biomass communities could be a contamination from other tissues and reagents was carefully evaluated and excluded. We also provide preliminary evidence that a disease-specific microbial signature characterized the intraocular environment of patients with age-related macular degeneration and glaucoma, suggesting that either spontaneous or pathogenic bacterial translocation may be associated with these common sight-threatening conditions. Furthermore, we revealed the presence of an intraocular microbiome in normal eyes from non-human mammals and demonstrated that this varied across species (rat, rabbit, pig, and macaque) and was established after birth. These findings represent the first-ever evidence of intraocular microbiota in humans.

## Introduction

Ocular health is central to the wellbeing of sighted mammals, and in humans up to half of our brain’s cortical neurons are devoted, directly or indirectly, to vision^1^. The factors responsible for maintaining a healthy eye are protean, and immunity is increasingly acknowledged to play a key role. This is despite the central nervous system’s immune privilege whereby inflammation is actively downregulated in the eye to preserve tissue integrity. In the context of disease, this privilege can be broken, and it has long been recognized that active immune responses cause retinal damage in conditions such as uveitis. However, their key role in blinding diseases where there is no clinically observable inflammation, such as age-related macular degeneration (AMD) and glaucoma, has only been clearly demonstrated recently and is now widely accepted^2^.

Concurrently, the field of immunology has exploded with evidence that microbes fundamentally shape immunity in mammals. This is most definitive in the setting of the gut microbiota, differences in which have clearly been shown to directly contribute to altered adaptive immune responses, with major implications for our understanding of inflammatory diseases across organ systems^3^. However, emerging reports are also starting to highlight that microbes translocate from the alimentary tract to seed disparate body sites^4^, most notably in experimental rodent models, which in the context of HLA-B27-associated spondyloarthropathy also includes the eye^5^.

The challenge of these observations is to understand their biological importance in both health and disease. It is not known whether these scant microbial populations interface with host tissues, or even whether they are alive. However, confirmation of their presence has the potential to be paradigm shifting in our consideration of host–microbe interactions in internal organs which were previously considered sterile, such as inside the eye^6^.

In this work, we present a structured analysis of the microbial content of fluid taken from the anterior chamber of both human and non-human mammalian eyes. First, we prospectively evaluated surgically obtained aqueous humor (AH) specimens from 1000 patients, which confirmed the presence of *Propionibacterium acnes* (*P. acnes*) in the majority of cases and suggested a diverse intraocular microbiota. Second, we characterized this diversity using metagenomic sequencing techniques. Third, we conducted exhaustive controls to minimize the risk that our observations were the result of contamination. Fourth, we compared the intraocular microbiome of patients with AMD and glaucoma with unaffected eyes, and finally we interrogated the intraocular microbial signatures of four non-human mammalian species (rat, rabbit, pig, and macaque) and found that the intraocular microbiota was established after birth in rats. We conclude that there is a low-biomass microbiome in what was previously considered to be a sterile intraocular environment and provide evidence that this is altered both in the context of disease and across mammalian species.

## Results

### Prospective cohort of over 1000 living human eyes

**Fig. 1:**
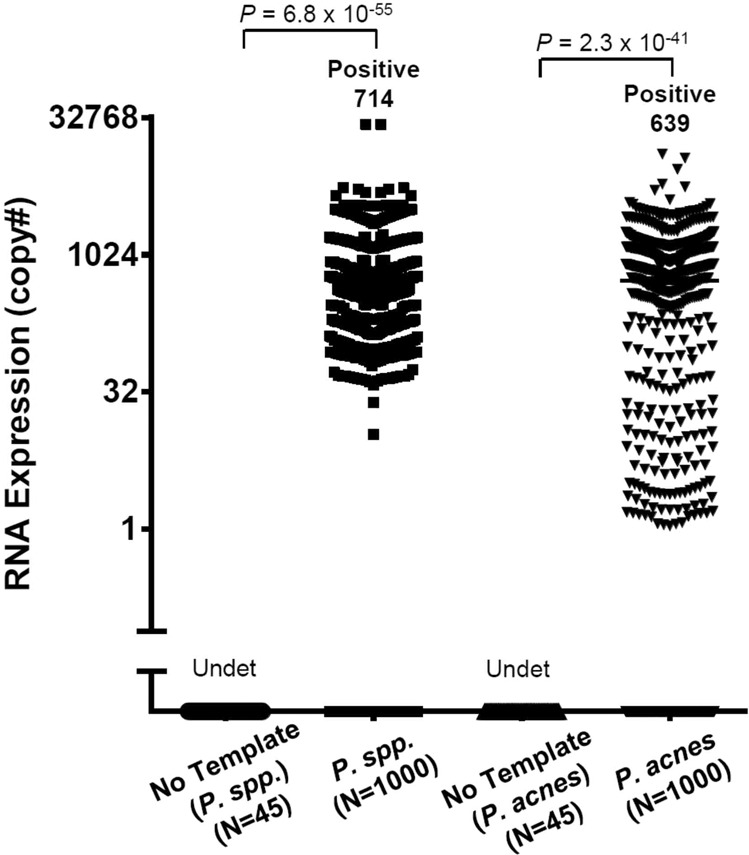
Detection of *P. spp*. in human AH specimens. Expression of *P. spp*. 16S rRNA and a *P. acnes*-specific gene—*PPA_RS04200* (presented as the copy#) in the AH from 1000 human eyes undergoing cataract surgery was quantified using real-time PCR assays. The *P* value was calculated using parametric Student’s *t*-test. Undet, undetected.

Our previous metagenomic and metatranscriptomic analysis of AH specimens from a patient with uveitis identified *P. acnes* as the most abundant bacterium inside the eye^6^. We therefore obtained 1000 specimens from eyes undergoing cataract surgery in patients without a history of intraocular inflammation, infection, or other ocular disease (Cohort1, Supplementary Table S1). These were tested for the RNA of *P. acnes* (one of the most common bacteria detected in chronically inflamed eyes after cataract operations), which confirmed 16S rRNA expression of *Propionibacterium spp*. (*P. spp.*) in 71.4% of eyes and RNA expression of a *P. acnes*-specific gene—*PPA_RS04200* in 63.9% of eyes, based on the real-time PCR assays (Fig. 1). We therefore conclude from this large prospective cohort that *P. acnes* RNA can be detected in the majority of anterior chamber samples from patients undergoing cataract surgery.

**Fig. 2:**
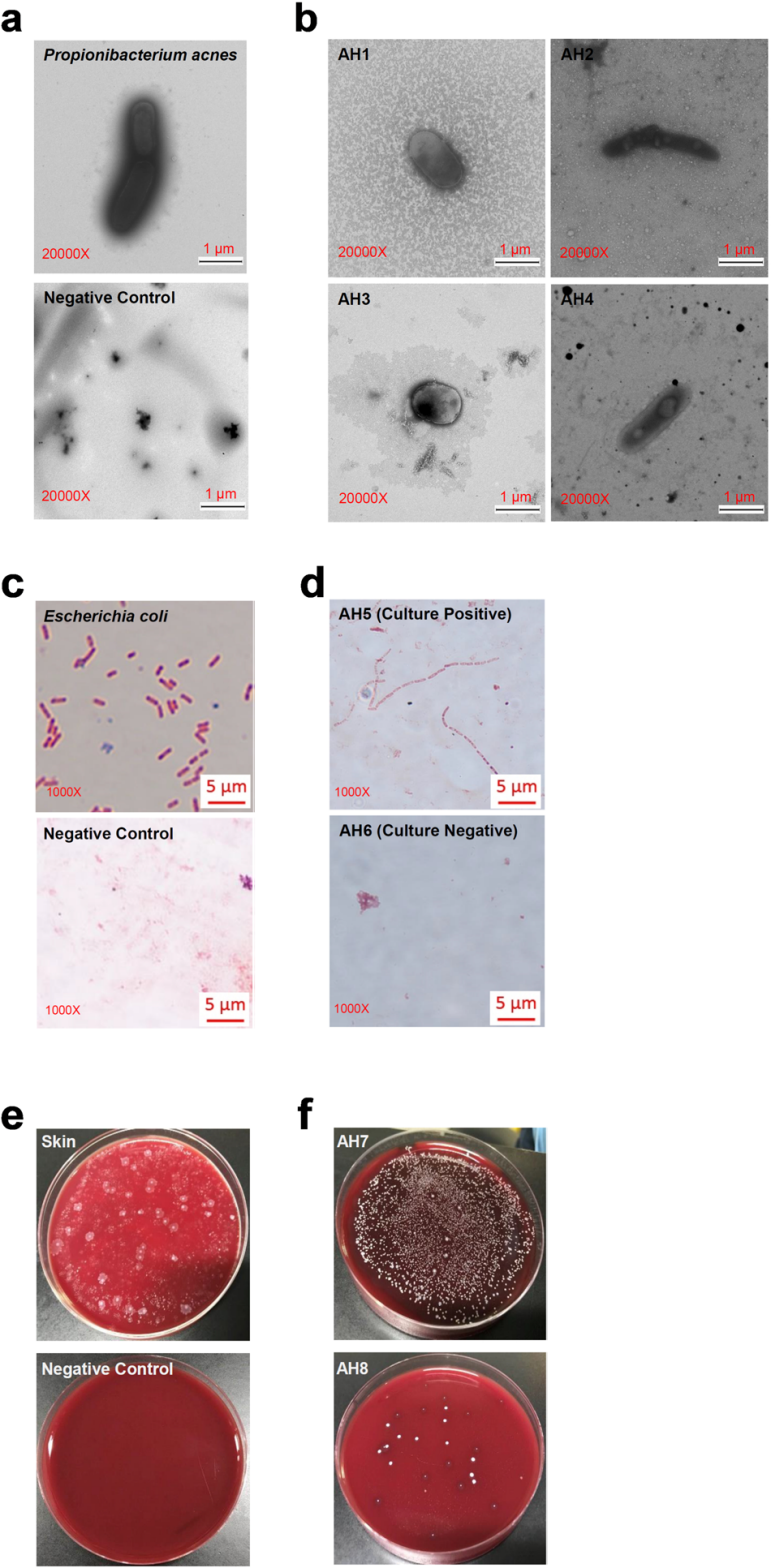
Detection of intraocular bacteria in AH specimens and cultures. **a** Negative staining transmission electron microscopy was used to visualize cultured *P. acnes* at 20,000× magnification. The negative control shows the visualization of water without AH specimen. **b** Negative staining transmission electron microscopy shows bacteria in minimally manipulated fresh AH specimens at 20,000× magnification. **c** Cultured *E. coli* was visualized by light microscopy. The negative control consists of sample preparation buffer without any AH inoculation. **d** Bacteria in cultured AH samples (examples of culture-positive and -negative samples) were visualized by light microscopy. **e** Representative anaerobic cultures of skin specimens and negative controls (PBS solution used to dilute specimens). **f** Representative anaerobic cultures of AH specimens.

This identification of intraocular *P. acnes* led us to ask whether it is possible to directly visualize these bacteria in intraocular fluid. AH specimens were examined using negative staining transmission electron microscopy. As a positive control, the rod-shaped cultured *P. acnes* was successfully visualized (Fig. 2a). In negative controls containing no AH specimens, no bacterium could be found using the identical negative staining protocol (Fig. 2a). However, multiple round- and rod-shaped bacteria were found in the AH samples from patients (Fig. 2b; Supplementary Fig. S1a). Endospores were evident within some bacteria (Fig. 2b) and free endospores were also found in AH specimens (Supplementary Fig. S1b). Hence, this indicated the existence of multiple types of intraocular bacteria in the AH specimens of cataract patients.

It is important to know whether the intraocular bacteria observed under microscope can be cultured. We therefore made multiple attempts to culture out the visualized bacteria from AH samples. Using five agar-based culture medium plates with various nutrients, we found no positive cultures of AH samples from cataract patients in aerobic cultures (Supplementary Fig. S2a), however cultures using liquid cooked meat medium covered by liquid paraffin wax, which resulted in hypoxia environment (Supplementary Fig. S2b), grew bacteria that could be visualized using standard light microscopes (Fig. 2c, d). Furthermore, positive anaerobic cultures of AH samples were found in cultures using blood plates (Fig. 2e, f). Among these bacterial clones resulting from the anaerobic cultures (with blood plates) of 10 AH specimens, we determined the identity of 20 clones using high-throughput sequencing technology. Interestingly, 13 out of 20 clones were identified as *P. acnes*, while *E*nterococcus *faecalis* (5 clones) and *Staphylococcus* *epidermidis* (2 clones) were the only two species identified in anaerobic cultures of AH samples (Supplementary Fig. S2c). Cultured bacteria from these AH samples were also examined using negative staining transmission electron microscopy. Both round- and rod-shaped bacteria were found (Supplementary Fig. S2d, e). We therefore concluded from these data that the viable intraocular bacterial community extends beyond *P. acnes*.

### Metagenomic analysis of human intraocular fluid

To characterize the diversity of microbial species in human eyes we recruited an independent cohort of 41 patients undergoing cataract surgery (Cohort 2, Supplementary Table S1) and subjected their AH samples to metagenomic analysis. This revealed a large number of human reads and the major kingdom of microorganisms was bacteria. Among all the 134 bacterial species found in at least one AH sample (Supplementary Table S2), *P. acnes* was the most abundant one found in the intraocular fluid (Supplementary Fig. S3). However, 12 bacterial species with at least average relative abundance of 1% were also found intraocularly (Supplementary Fig. S3), suggesting that a possible complex community of bacteria might be present inside the eyes. In addition to the detection of *P. spp*.-transcribed RNA in the eyes of most cataract patients (Fig. 1), our metagenomic data also demonstrated that sequencing reads of *P. acnes* from individual AH samples were able to cover the full-length genome of the organism (Supplementary Fig. S4) and were not from fragmented pieces of DNA that was potentially introduced into specimens during AH sampling.

### Further control studies to minimize the risk of a false-positive result due to bacterial contamination

**Fig. 3:**
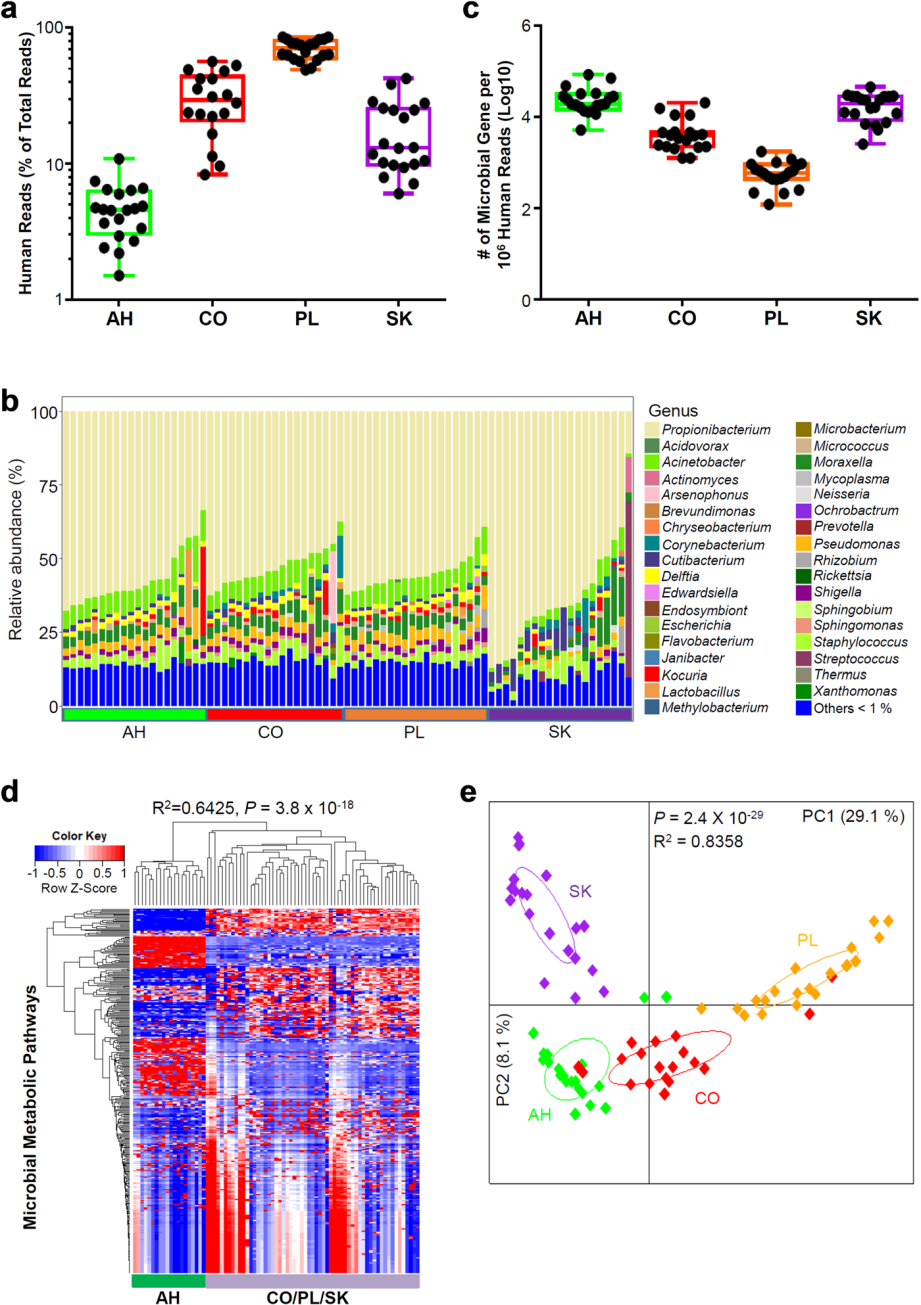
A unique intraocular microbiota detected by metagenomic sequencing analysis. Comparative metagenomic analysis was performed for AH, conjunctiva (CO), plasma (PL), and skin (SK) specimens from 20 patients undergoing cataract surgery. **a** The average percentage of human reads among total sequenced reads from AH, CO, PL, and SK samples. **b** Relative abundance of the major genera of bacteria in the metagenomes of AH, CO, PL, and SK samples. **c** The average numbers of microbial genes identified in AH, CO, PL, and SK samples. **d** Hierarchical clustering analysis of relative abundance of microbial metabolic pathways identified in AH, CO, PL, and SK samples. *P* value was calculated using ANOVA test. **e** Diversity of the microbial community analyzed by PCoA. The Bray Curtis distance was used and *P* value was calculated using PERMANOVA test.

In order to address the possibility that our results were secondary to contamination from periocular tissues or bleeding at the time of surgery, we performed a metagenomic comparison of simultaneously acquired specimens of AH, conjunctiva, eyelid skin, and plasma from a further 20 patients undergoing cataract surgery (the demographic characteristics of all patients were listed in Cohort 3, Supplementary Table S1). A large number of human reads were detected among samples from all tissues (Fig. 3a); however, the number of human reads within the AH samples (~ 5%) was significantly lower than the other three tissues (Fig. 3a). The bacterial community in the specimens from all tissues showed classical individuality (Fig. 3b and Supplementary Fig. S5a). The average numbers of microbial genes detected in AH samples (Fig. 3c) as well as the alpha diversity (detected by Shannon index) of the microbial community in AH (Supplementary Fig. S5b) were significantly higher than in the conjunctiva (CO) and plasma (PL) samples. Unsupervised hierarchical clustering of metabolic pathways in microbiota from the four tissues uncovered a unique metabolic pattern enriched in AH samples that was distinct from the other three tissues, which were indistinguishable (Fig. 3d and Supplementary Table S3). A principal coordinate analysis (PCoA) of community similarity (including bacteria, fungi, and viruses) indicated that the metagenome of AH was different from the other three tissues (Fig. 3e and Supplementary Fig. S6a–c). The relative abundance of fungal and viral species differed among the four tissues (Supplementary Fig. S7).

These data suggest that the bacteria detected in the AH specimens are not contaminations from other tissues. However, to further rule out the possibility that the bacteria found in our AH specimens were contaminations from other tissues involved in AH sampling, we went on to test our method of eye disinfection at the time of cataract surgery by applying the same procedure to 8 macaques (16 eyes) and compared the total bacterial abundance on the inferior bulbar conjunctiva before and after the disinfection procedure. In addition, PCR assays capable of detecting all bacterial 16S rDNA were also performed on the reagents and drugs used during the sampling process. Data shown in Supplementary Fig. S8a, b demonstrated that our disinfection procedure abolished bacterial species on the surface of most eyes.

Potential reagent and environmental contamination in high-throughput sequencing experiments was also a concern for our study. We thus paid great attention to myriad negative controls for both environments and reagents throughout these studies, including the blank, wash solution, anesthetic, disinfectant, NaCl solution, and mydriatic samples. Eight independent samples (blank) were collected following the exact procedures for AH collection, substituting 100 μL 0.9% sodium chloride solution instead of AH into the sample collection tubes in the operation room. Similarly, two of each wash solution, anesthetic, disinfectant, NaCl solution, and mydriatic samples were collected as controls for metagenomic sequencing analysis of AH specimens. All tissue samples as well as environmental and reagent controls were analyzed using a metagenomic sequencing approach. We found no detectable DNA in all control samples, while an average of 0.13 ng/μL DNA could be detected in AH samples using Qubit (Life, USA) (Supplementary Fig. S9a). Importantly, all negative control samples required at least 40 cycles of PCR amplification in order to obtain enough DNA for sequencing experiments (Supplementary Fig. S9b). As a result, over amplified DNA in negative control samples resulted in DNA bands on gels that were different from the AH samples (Supplementary Fig. S9b). These data clearly differentiate the controls from AH samples, and this validation process confirms that the reads from AH samples are indeed from a true metagenomic community.

To further address the concerns of contamination in the metagenomic analysis of low-biomass specimens such as AH, we obtained additional 12 AH specimens (Supplementary Table S1, Cohort 4) and performed a spike-in (0.1 ng *Malassezia globose* genome, MYA4612 from ATCC, USA) controlled comparative analysis of AH metagenomes using two commercially available DNA extraction kits. Twelve AH specimens were divided into two equal parts, which were subjected to DNA isolation using QIAamp PowerFecal DNA kit (Qiagen, USA) (named group Q) and Epicentre MasterPure Complete DNA and RNA Purification Kit (Epicentre, USA) (named group E), respectively. Three blank samples without AH specimens were processed along the metagenomic sequencing analysis and served as negative controls (named Q-blank and E-blank, respectively) and any species present in these blank controls were excluded from further analysis of AH samples (Supplementary Table S4). As shown in Supplementary Fig. S10a, the alpha diversities measured by Shannon index of bacterial communities in Q and E groups were comparable, which were significantly higher than the alpha diversities of the communities in both blank controls. Although blank controls from Q and E groups were very different in beta diversity, beta diversities of the bacterial communities in Q and E groups were similar (Supplementary Fig. S10b). When the relative abundance of bacterial species was compared (Pearson correlation coefficient) between communities identified using the Q or E kits, we found highly correlated communities identified in 11 out of 12 sample pairs (R ranged between 0.70 and 0.96) (Supplementary Fig. S10c). Within the bacterial communities, the majority of core species with the relative abundance above 1% were consistently identified by both DNA isolation kits (Supplementary Fig. S10d). In particular, *P. acnes* was the most abundant bacterial species within all samples (Supplementary Fig. S10e). Its relative abundance (in average) was almost the same in groups Q and E samples (Supplementary Fig. S10d, top right corner). These results demonstrated that the microbial communities we identified from AH specimens were not a contamination from reagents or environments.

### The intraocular microbial signature of patients with age-related macular degeneration and glaucoma

**Fig. 4:**
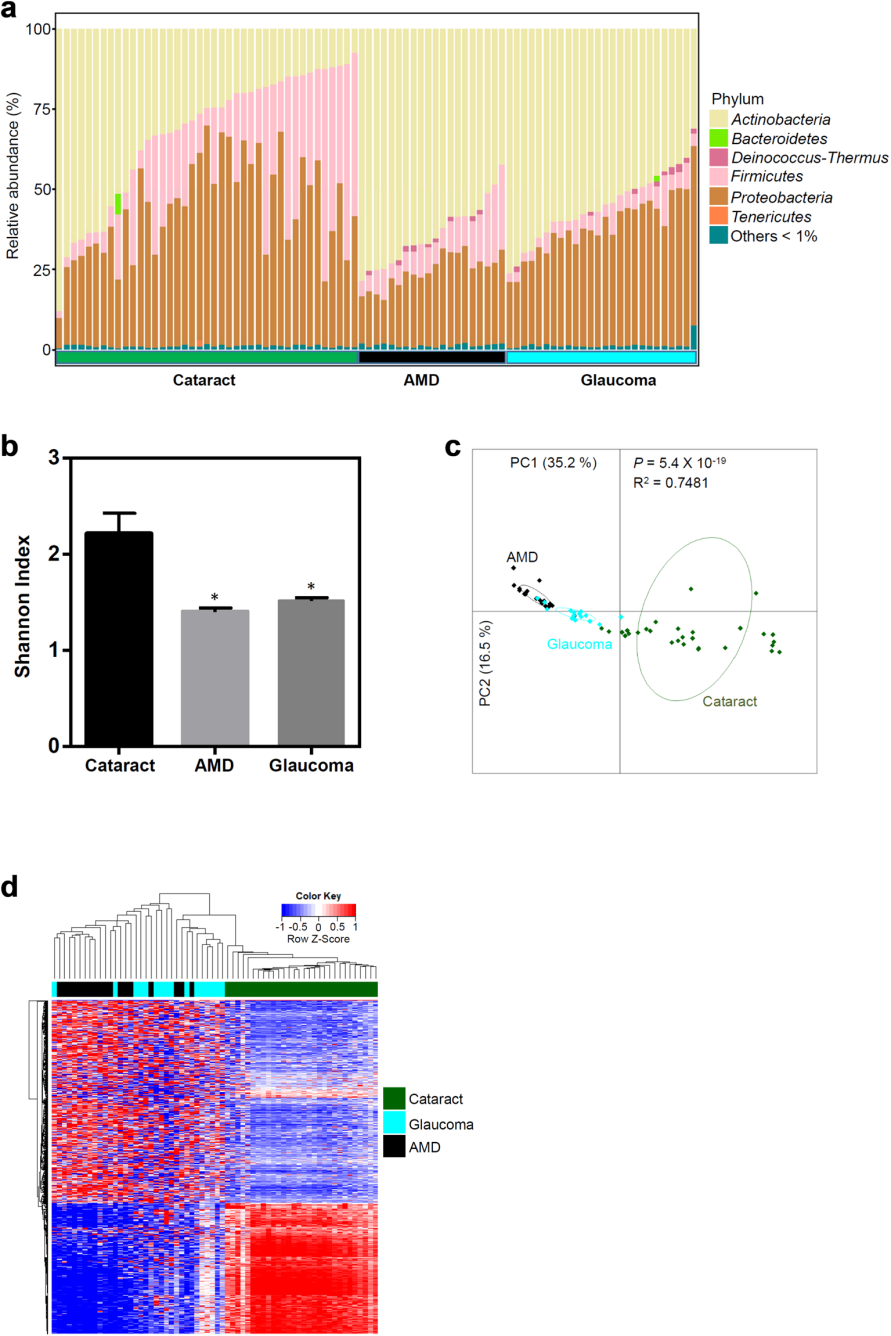
The microbiota in AH from patients with cataract, AMD, and glaucoma. **a** Relative abundance of the six major phyla of bacteria in the intraocular metagenomes in patients with cataract (*N* = 41), AMD (*N* = 20), or glaucoma (*N* = 26). **b** The alpha diversity (measured by Shannon index) of the intraocular metagenomes in patients with cataract, AMD, and glaucoma. The error bar represents the mean of all Shannon index within the disease group ± SEM. The statistical difference was measured between patients with cataract and each of other disease groups, respectively. Mann-Whitney *U* test significance levels are denoted by asterisks (*P < 0.05). **c** PCoA of the similarity of the intraocular metagenomes in patients with cataract (*N* = 30), AMD (*N* = 18), and glaucoma (*N* = 16). The Bray Curtis distance was used and *P* value was calculated using PERMANOVA test. **d** Hierarchical clustering analysis of the functional genes in the intraocular metagenomes in patients with cataract (*N* = 30), AMD (*N* = 18), and glaucoma (*N* = 16).

In order to define the intraocular microbiome in the context of disease, we conducted a preliminary comparison of the metagenomic sequencing results from our group of 41 otherwise normal eyes undergoing cataract surgery (Cohort 2) with an additional 38 patients who had a diagnosis of AMD (*N* = 20) (Cohort 5) or glaucoma (*N* = 26) (Cohort 6) at the time of cataract surgery. Supplementary Table S1 listed the summary demographic characteristics of all patients. The bacterial community in the AH specimens from all three patient cohorts showed classical individuality (Fig. 4a, Supplementary Fig. S11a, and Table S5), similar to the microbiome found in other body parts. Interestingly, the alpha diversities of the intraocular microbial communities were significantly different among these three types of patients (Fig. 4b), despite all patients having bacteria as the major component of their intraocular microbiome (Supplementary Fig. S11b–d). The PCoA on the composition of the intraocular microbiota (using all microbial species) showed clear differences among cataract, AMD and glaucoma patients (Fig. 4c and Supplementary Fig. S12a, b). Importantly, the intraocular microbial communities in all patients were significantly different from all specimen-free negative controls (Supplementary Fig. S13). Similarly, hierarchical clustering analysis of the relative abundance of functional microbial genes from all metagenomes indicated that each ocular manifestation had a general signature of microbial function, while there were outliers in AMD or glaucoma group that could be classified to the other disease clusters (Fig. 4d). In spite of the significant individuality presented by the intraocular microbiome (Fig. 4a), we were able to identify signature bacterial species for each ocular disease group we tested (Supplementary Fig. S14). Taken together, our results suggest that the composition and function of intraocular microbiota may differentiate certain ocular diseases.

### Metagenomic analysis of AH specimens across non-human mammalian species

**Fig. 5:**
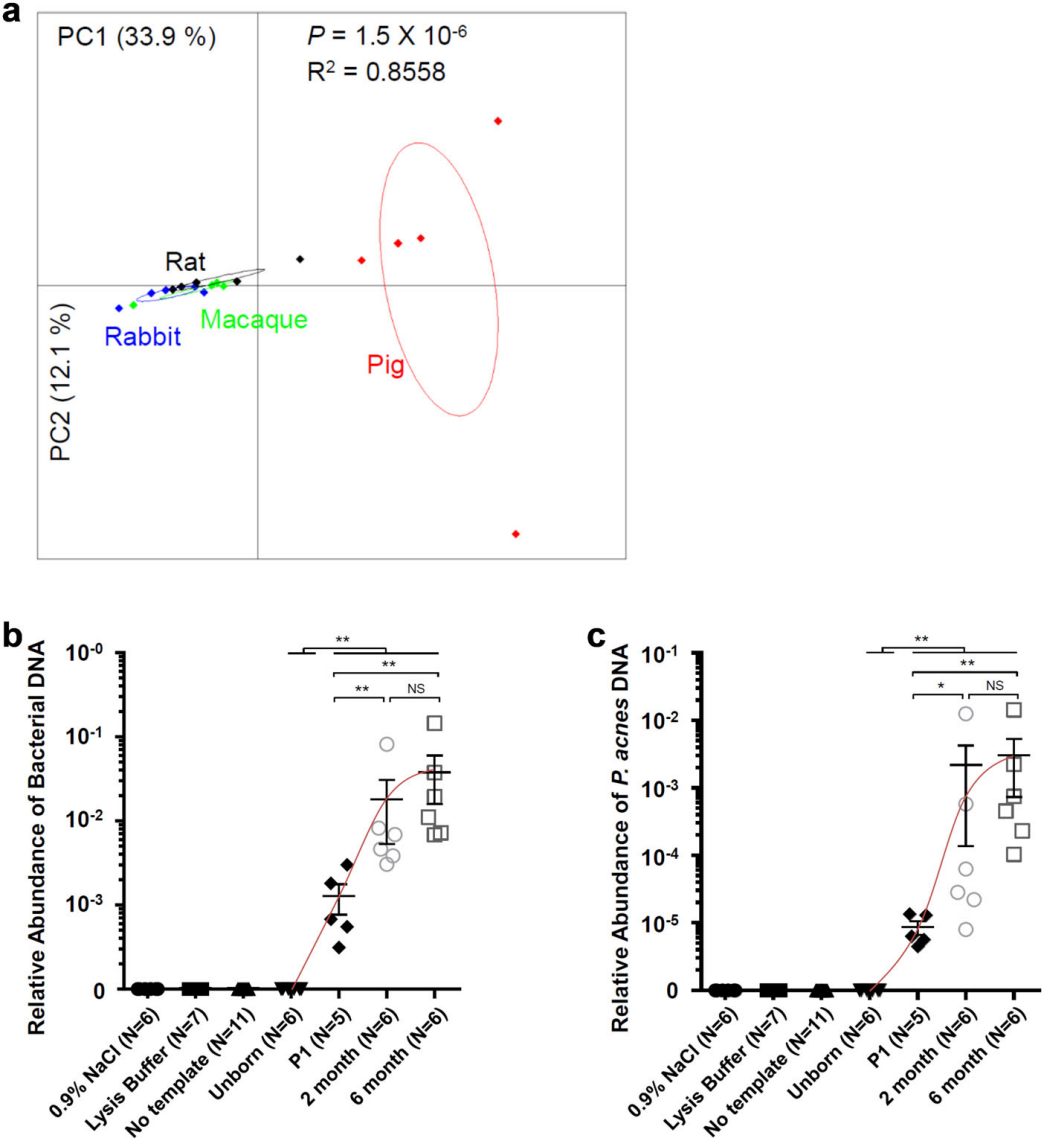
The intraocular metagenome in the eyes of rabbit, rat, macaque, and pig analyzed using PCoA. **a** The PCoA of intraocular metagenomes in four mammals. The Bray Curtis distance was used and *P* value was calculated using PERMANOVA test. **b** The abundance of bacterial DNA in rat intraocular fluids was measured using real-time PCR assays of total 16S rDNA and represented as the relative abundance to the level of rat *Actb* gene. **c** The abundance of *P. acnes* DNA in rat intraocular fluids was measured using real-time PCR assays of *P. acnes* 16S rDNA and represented as the relative abundance to the level of rat *Actb* gene. The error bar represents the mean ± SEM. The statistical difference was measured using Mann-Whitney *U* test and the significance levels are denoted by asterisks (*P < 0.05, **P < 0.01, NS: not significant).

Finally, we obtained AH samples from healthy rat (*Rattus norvegicus*), rabbit (*Oryctolagus cuniculus*), pig (*Sus scrofa*), and macaque (*Macaca fascicularis*) eyes under sterile laminar flow conditions. Metagenomic analysis of these 20 AH specimens confirmed a genomic signature consistent with a diverse microbial population in all four species (Fig. 5 and Supplementary Fig. S15a, b). In addition, our data indicated that the profiles of AH metagenomes in rat, rabbit, and macaque differed substantially from pigs (Fig. 5a). One important question is when the intraocular microbiota is established during life span. We therefore obtained intraocular fluids (pooled aqueous and vitreous humors from each eye) from rat eyes at different stages of life (3 animals and 6 eyes per stage), including unborn (delivered by cesarean section), 1-day-old, 2-month-old, and 6-month-old. The abundance of total bacterial DNA and *P. acnes* DNA was measured using real-time PCR assays of 16S rDNA and represented as the relative abundance to the host *Actb* gene. Interestingly, neither total bacterial DNA nor *P. acnes* DNA was detectable in the eyes from unborn rats, while they were found in the intraocular fluids after birth and reached a relative stable level in adult rats (Fig. 5b, c). Taken together with our human data, these results demonstrate that intraocular bacterial DNA can be detected across a range of mammalian species.

## Discussion

In the past 10 years, the diversity and function of microbiota associated with human health and disease have been extensively studied through high-throughput sequencing technologies and microbiomic/metagenomic analysis^7^. The local microbiota of the eye under physiological and pathological conditions remains largely uncharacterized^8,9^, and the theory that the intraocular cavity is absolutely sterile under physiological conditions has led many researchers to reason that any types of foreign organisms are exogenous and pathogenic.

In our study: (1) by using the quantitative PCR technique and a large cohort of patients, we found RNA of *P. acnes* in 64%–71% AH specimens from uninflamed eyes (Fig. 1), which was not from the contamination of ocular surface during sampling of the AH (Supplementary Fig. S8); (2) by using electron microscopy, we visualized multiple types of bacteria in AH specimens (Fig. 2b); (3) by using various microbial culture techniques, we found ~ 93% AH were positive for multiple types of bacteria, which were clearly different from the uncultured intraocular bacteria in terms of morphology and activity (Fig. 2); (4) by using the metagenomic sequencing technology, we were able to distinguish the composition of AH from conjunctival, plasma, and skin microbiota (Fig. 3); (5) we provide preliminary evidence that the complexity and signatures of intraocular microbiota might also characterize ocular diseases such as glaucoma, or AMD (Fig. 4); (6) by using metagenomic sequencing technology we found bacterial DNA inside of the eyes of four disparate non-human mammalian species (Fig. 5a); and (7) similar to the gut microbiota^10,11^, we found that the intraocular microbiota was established after birth in rats, increased in its abundance during life, and reached the highest level in adults (Fig. 5b, c).

A crucial premise of the human data we have presented is that AH samples taken at the start of cataract surgery are representative of the normal intraocular environment. This is consistent with the longstanding practice in vision research of using intraocular samples taken at the time of cataract surgery as control materials for comparison with eyes affected by various other ocular pathologies. Our data therefore indicate that even eyes with no signs of ocular distress or infection have an individualized microbiome with compositional and functional diversity.

Culture-independent sequencing-based technologies are extremely sensitive and powerful for characterizing many microbial communities in health and disease^12^. However, these microbiome and/or metagenomic studies also discovered controversial microbiota in the body sites or environments that were previously considered sterile, for example, placenta^13,14^. Actually, contamination in low microbial biomass microbiome studies could be introduced during sample collection and storage, laboratory processing of samples for sequencing experiments including reagents, and bioinformatic analysis steps^12,15,16^. Our current study integrated multiple key steps to control possible contamination and avoid false conclusions. First of all, we demonstrated that disinfection of ocular surface prevented introduction of bacterial contamination during sampling of intraocular AH specimens (Supplementary Fig. S8), which resulted in the characterization of intraocular microbiota that were different in both composition and functions from the ones in other tissues including skin, conjunctiva, and plasma (Fig. 3). Second, we applied direct visualization by electron microscopy (Fig. 2), PCR analysis (Fig. 1), in vitro culture (Fig. 2), as well as culture-independent sequencing techniques (Figs. 3–5) to avoid bias and erroneous conclusions reached by metagenomic analysis alone. Importantly, we also collected negative controls covering every step of the experimental process (Supplementary Figs. S8–S10, and S13) to minimize the possible misinterpretation of our data set. Finally, we also applied several controls in the bioinformatic analysis pipelines in order to limit false discoveries from our metagenomic analysis.

Recent reports have demonstrated translocation of gut commensals to the bloodstream^17^, liver^4^, pancreas^18^, and joints^5^ in animals and patients. In addition, tumor-type-specific intracellular bacteria were also detected in multiple human tumors with low microbial biomass^19,20^. Although the mechanisms of microbial translocation remain elusive, many factors influencing this process have been proposed^21^. Characteristics of both bacteria and the host decide the process of spontaneous or pathological translocation of whole bacteria into other internal organs. The host barriers including epithelial, vascular, and lymphatic ones restrict the passage of bacteria, whose integrity or selective gatekeeping property may regulate the traffic of free bacteria and/or host cells loaded with intracellular bacteria. Certain bacteria may also survive in the autophagosomes within phagocytes like Trojan horses and migrate with these cells through barriers, from gut to body sites including the eye and skin^22,23^. In addition, many commensal bacteria also likely co-evolve with the host and induce tissue-specific host immune tolerance to the dormant forms of these bacteria in the eye^24^. However, it is feasible that the intracellular location of these bacteria could be altered in response to pathogenic factors, which in theory would be consistent with the concept of dysbiosis of tissue-specific microbiota in eye diseases, hence our findings may have novel relevance to the pathogenesis of a range of sight-threatening conditions.

Several types of intraocular cells, including retinal pigment epithelium, may process and present foreign peptides through MHC class I molecules on their surface without immediately inducing a functional immune response. Once a breakdown of tolerance and/or physical vascular barriers occurs, specialized immune responses against the pathogenic microbes would then have the potential to initiate and target the ocular cells presenting the foreign antigens on their surface. However, the question of how commensal microbes gain entrance to the intraocular space and whether they subsequently influence health and disease is yet to be answered.

All techniques have limitations and bias. More positive and negative controls could be used in order to even more exhaustively limit the possible introduction of microbial contamination into the intraocular specimens. In addition, we recognize the potential that eyes with cataract might not be truly ‘normal’, therefore, additional procedure- and disease-free eyes may be needed for further confirmation of intraocular microbial community.

In conclusion, using electron microscopy, PCR, culture, and metagenomic sequencing analysis, we detect the presence of a bacterial community in AH samples taken from multiple human donors, with or without associated ocular pathology. This observation was further replicated in other mammalian species, suggesting that a low-biomass intraocular microbiome is a hitherto unrecognized component of the eye with potential wide-ranging implications for the future study of ophthalmic health and disease.

## Methods

### Subject recruitment

Patients with cataract (Cat), glaucoma (Gla), and AMD were recruited at Zhongshan Ophthalmic Center (Guangzhou, China) and Tianjin Medical University Eye Hospital (Tianjin, China) between September 2014 and August 2018. The basic demographic information for these 6 cohorts (1119 patients) was listed in Supplementary Table S1. This study adhered to the tenets of the Declaration of Helsinki and was approved by the Institutional Review Boards of Zhongshan Ophthalmic Center, Sun Yat-sen University (protocol #2014MEKY024, #2014MEKY032, and #2016KYPJ031) and Tianjin Medical University Eye Hospital (protocol #2016KY-14). All subjects provided written informed consent before participation.

### Sampling of AH from patients

A topical antimicrobial drug, 0.5% levofloxacin eye drops (Cravit, Santen Pharmaceutical Co., Japan), was administered four times a day in both eyes for at least 3 days before the cataract surgery. On the day of surgery, patients received conjunctival sac irrigation using 0.9% sodium chloride solution at least twice and mydriasis using compound tropicamide eye drops. Following disinfection and draping, 5% povidone iodine (PVI) was applied on the eye for 30 s. The conjunctival sac was then irrigated with tobramycin solution for at least three times. After topical anesthesia with 0.5% alcaine (at least three times), auxiliary incision was performed using 1.5 mm stab knife (Alcon, USA) at the 2 o’clock position of the limbus. AH was sampled via the auxiliary incision using a 1 mL sterile syringe before any other procedures were initiated. Immediately after collection, AH samples were transferred into sterile eppendorf tubes and stored at −80 °C prior to DNA extraction. The 0.9% sodium chloride solution (100 μL) was also transferred from a fresh 1 mL sterile syringe into a sterile eppendorf tube, which served as the Blank control.

### Sampling of AH from animal eyes and detection of total bacterial DNA using real-time PCR assays

The eye balls from animals free of diseases and genetic manipulation were sterilized using 5% PVI and tobramycin solution, followed by washing in sterile 0.9% sodium chloride solution for three times in a cell culture hood. AH was sampled using 1 mL sterile syringes. DNA extraction was carried out using MasterPure Complete DNA and RNA Purification Kit (Epicentre, USA) according to the manufacturer’s protocol. The total bacterial DNA relative to the host DNA was quantified using the primer pair previously used in Clifford et al.^25^, detecting 16S rDNA of all bacteria: U16SRT-F: ACTCCTACGGGAGGCAGCAGT; U16SRT-R: TATTACCGCGGCTGCTGGC.

### Sampling of plasma

Approximately 5 mL of peripheral venous blood was collected in an EDTA-anticoagulated vacutainer tube and then centrifuged for 10 min at 2000 rpm. The supernatant was collected and stored at –80 °C prior to analysis.

### Sampling of conjunctiva and detection of total conjunctival bacterial DNA using PCR assays

The conjunctival impression cytology samples from inferior bulbar conjunctiva were obtained using the following protocol: (1) topically anesthetize the eye with 1–2 drops of Alcaine Eye Drop (Alcon, USA) and keep the eye closed for several minutes; (2) using disposable tweezers, place the MF Membrane filter (Millipore, REF:HAWP01300, 0.45 μm) on the inferior bulbar conjunctiva with the edge of the membrane clear of the lower tear meniscus and gently press for 10–15 s; (3) remove the membrane and store it immediately at –80 °C in a sterile Eppendorf tube with 300 μL Tissue and Cell Lysis Solution containing 1 μL of Proteinase K, provided in the MasterPure Complete DNA and RNA Purification Kit (Epicentre, USA); (4) apply 1–2 drops of Neomycin Sulfate eye drops (Alcon, USA) to each examined eye. DNA extraction was carried out using MasterPure Complete DNA and RNA Purification Kit (Epicentre, USA) according to the manufacturer’s protocol from the conjunctival impression cytology samples. The total bacterial DNA was PCR amplified using the primer pair previously used in Oh et al.^26^, detecting 16S rDNA of all bacteria (the amplicon at the size ~ 530 bp is between V1 and V3): V1_27F: AGAGTTTGATCCTGGCTCAG; V3_534R: GCATTACCGCGGCTGCTGG.

### Sampling of eyelid skin

Facial skin specimens were collected by scraping the skin of lower eyelid with a sterile MF Membrane filter (Millipore, REF:HAWP01300, 0.45 μm). The sample was inserted into a sterile eppendorf tube with 300 μL lysis solution (Epicentre).

### RNA extraction and real-time PCR

Total RNA was extracted from 100 μL AH using MasterPure Complete DNA & RNA Purification Kit (Epicentre, USA). DNA-free RNA was reverse transcribed into DNA using the All-In-One Master Mix Kit (Kapa, USA). Real-time PCR was performed using KAPA SYBR FAST Universal qPCR kit (Kapa, USA) to quantify the copy numbers of bacterial RNA. The primers were used: *P. spp.*-*16S*: F-CCTGCCCTTGACTTTGG, R-AAGCCGCGAGTCCATC; *P. acnes*-*PPA_RS04200*: F-GATTGGTTTACTACCCGTGAGCG, R-ATAGCAGGGATTCCA-GCGACA.

### DNA extraction from AH, plasma, conjunctiva, and skin specimens

DNA extraction was carried out using MasterPure Complete DNA and RNA Purification Kit (Epicentre, USA) or QIAamp PowerFecal DNA kit (Qiagen, USA) according to the manufacturer’s protocol.

### Intraocular bacterial culture

#### Aerobic culture in solid medium

The following agar mediums purchased from Huankai Microbial Inc. (Guangzhou, China) were sterilized at 121 °C for 30 min and used to culture AH samples at 37 °C for 5 days in a HettCube 200 incubator (Germany):

Brain-Heart Infusion Agar Medium (BHI Agar), pH 7.2: 12.5 g/L brain infusion solids, 5 g/L beef heart infusion solids, 10 g/L proteose peptone, 2 g/L glucose, 5 g/L NaCl, 2.5 g/L Na_2_HPO_4_, 15 g/L agar.

Soybean-Casein Digest Agar Medium (TSA Agar), pH 7.2: 15 g/L tryptone, 5 g/L soytone, 5 g/L NaCl, 15 g/L agar.

Nutrient Agar 1 (N1 Agar), pH 7.2: 1 g/L Lab-Lemco’ powder, 2 g/L yeast extract, 5 g/L peptone, 5 g/L NaCl, 15 g/L agar.

Nutrient Agar 2 (N2 Agar), pH 7.2: 10 g/L beef extract, 10 g/L tryptone, 5 g/L NaCl, 3 g/L yeast extract, 3 g/L CH_3_COONa, 1 g/L soluble starch, 0.5 g/L L-cysteine hydrochloride, 15 g/L agar.

Nutrient Agar 3 (N3 Agar), pH 7.2: 10 g/L peptone, 3 g/L beef extract, 5 g/L NaCl, 15 g/L agar.

#### Hypoxia culture in liquid medium

The cooked meat medium (without antibiotics, 5 g/L Lab-Lemco’ powder, 30 g/L peptone, 5 g/L yeast extract, 5 g/L NaH_2_PO_4_, 3 g/L glucose, and 2 g/L soluble starch) was purchased from Huankai Microbial Inc. (Guangzhou, China). Each culture was prepared in a 15 mL glass tube (purchased from Drtech Inc., Guangzhou China) with 6 mL cooked meat medium, sterilized dry beef granules, and 1.5 mL liquid paraffin wax (purchased from Huankai Microbial Inc., Guangzhou, China) on top. All tubes were then sterilized at 121 °C for 30 min in the HEV-to Autoclave instrument (HIRAYAMA, Japan). AH sample fluid was injected into above tube in sterilized cell culture hood and sealed, followed by culture with shaking (200 rpm) at 37 °C for 5 days in the ZQTY-70F incubator (Zhichu Instrument Co., Ltd, Shanghai, China). Wax-sealed tubes containing no AH sample but the culture medium underwent the incubation protocol served as the negative controls. All cultures were then gram stained and subjected to microscopic examination.

#### Anaerobic culture in blood agar medium

Each AH specimen was mixed with sterile PBS to make a 200 μL mixture, was inoculated onto blood agar plate (purchased from CRmicrobio, Guangzhou, China), and cultured anaerobically in the incubator (LHS-50CL, Shanghai, China) at 37 °C for 7 days. Anaerobic environment was generated using atmosphere generation systems AnaeroGen^TM^ 2.5 L (ThermoFisher, USA). The sterile PBS was used as negative controls.

Blood agar medium: 10.0 g/L peptone, 3.0 g/L Cardiac trypsin digestion, 1.0 g/L corn starch, 15.0 g/L agar, 5.0 g/L meat stomach enzyme digestion, 5.0 g/L yeast extract, 5.0 g/L NaCl, 70 mL/L sheep blood, ddH_2_O.

### Negative staining transmission electron microscopy

Fifty µL of fresh AH specimens or cultured AH samples were centrifuged at 14,000 rpm for 20 min. Supernatant was removed saving 5 µL of fluid, which was then loaded onto a copper grid with carbon film. The grid with sample was then stained with 2 µL phosphotungstic acid (3%) for 1 min. The grid was immediately examined using a JEM2010 electron microscope (JEOL Ltd., Japan). The images were acquired on a 2k × 2k 895 CCD camera (Gatan, CA USA). All reagents and grids were sterilized. Water without any AH samples was used as negative controls and no bacteria were found after extensive search in negative controls.

### Metagenomic sequencing

A total of 100 ng of DNA from each sample was sonicated into fragments of 300–400 bp using Bioruptor (Diagenode, Belgium) and subjected to sequencing library preparation following the standard protocol provided by the manufacturer using VAHTS Nano DNA Library Prep Kit for Illumina (Vazyme, China). DNA libraries were sequenced to a depth of 10~50 million reads per sample using HiSeq PE Cluster Kit v4 and HiSeq SBS V4 250 cycle kit (Illumina, USA) on the Illumina HiSeq2500 sequencer and subjected to initial processing using CASAVA (v1.8.2) (Illumina).

### Metagenomic data analysis

Pre-processing of sequencing reads: All reads were first evaluated by FastQC for quality control. To maintain the consistency of alignment accuracy among all microbial reads, we first trimmed all reads to 75 bp using PrinSeq (v0.20.4)^27^, which provided a best Q30 in our sample set. Paired-end reads from each sample were combined into one single file and treated as single-end reads. Low-quality reads, replicated reads, and potential adapter sequences were removed using Fastx toolkit (v0.0.12). The reads containing more than 10% of ambiguous bases were depleted using PrinSeq (v0.20.4). Human reads were then removed from the subsequent analysis using HiSAT2 (v2.0.1)^28^, BMTagger, and DeconSeq^29^ to obtain clean non-human sequences.

#### Sequence analysis

The non-human sequences were first analyzed using the Kraken program^30^, with the pre-built 4 GB database as the reference (including complete bacterial, archaeal, and viral genomes in RefSeq as of 8 December 2014) (https://ccb.jhu.edu/software/kraken/), followed by mapping of non-human sequences against our custom fungal genomes (containing 68 species and 75 strains, downloaded from http://fungidb.org/fungidb/, on 12 March 2015) using Burrows-Wheeler Aligner (BWA0.7.5a) with three mismatches. The relative abundance of each species was calculated by the ratio of the total mapped reads of each species, normalized by their genome size and the total mapped microbial reads within each sample. Decontam^31^ (in R scripts) was used to remove all contaminations from negative sequencing controls. Community diversity (Shannon index and evenness) was calculated according to the method described in Mothur program after using a subsampling cutoff of 500 microbial sequences per sample. The HMP Unified Metabolic Analysis Network (HUMAnN2)^32^ was used to analyze the abundance of microbial genes and KEGG pathways for samples with >500 microbial sequences per sample. PCoA was performed on the relative abundance of bacterial species or microbial genes using Ade4 package in R statistical software (v3.1.1) after using a subsampling cutoff of 500 microbial sequences per sample or 1 read per gene. LDA Effect Size (LefSe 1.0)^33^ was used to identify species, microbial genes, and functional pathways characterizing the differences among sample groups.

### Supplementary information

Supplementary Figures

Supplementary Table S1

Supplementary Table S2

Supplementary Table S3

Supplementary Table S4

Supplementary Table S5
