# Author Correction: Identification of an intraocular microbiota

**DOI:** 10.1038/s41421-024-00675-y

**Published:** 2024-05-15

**Authors:** Yuhua Deng, Xiaofei Ge, Yan Li, Bin Zou, Xiaofeng Wen, Weirong Chen, Lin Lu, Meifen Zhang, Xiaomin Zhang, Chunmei Li, Chan Zhao, Xiaofeng Lin, Xiulan Zhang, Xinhua Huang, Xiaorong Li, Ming Jin, Guang-Hua Peng, Dongni Wang, Xun Wang, Weiyi Lai, Juanran Liang, Jing Jing Li, Qiaoxing Liang, Liu Yang, Qinfen Zhang, Yinyin Li, Ping Lu, Xiao Hu, Xifang Li, Xiuli Deng, Yu Liu, Yanli Zou, Shixin Guo, Tingting Chen, Yali Qin, Fuhua Yang, Li Miao, Wei Chen, Chi-Chao Chan, Haotian Lin, Yizhi Liu, Richard W. J. Lee, Lai Wei

**Affiliations:** 1 State Key Laboratory of Ophthalmology, Zhongshan Ophthalmic Center, Sun Yat-sen University, Guangzhou, Guangdong, 510060 China; 2 Department of Ophthalmology, Peking Union Medical College Hospital, Chinese Academy of Medical Sciences, Beijing, 100730 China; 3 Tianjin Medical University Eye Hospital, Eye Institute & School of Optometry and Ophthalmology, Tianjin, 300384 China; 4 Department of Ophthalmology, China-Japan Friendship Hospital, Beijing, 100029 China; 5 Department of Pathophysiology, Basic Medical College of Zhengzhou University, Zhengzhou, He’nan 450001 China; 6 Department of Ophthalmology, General Hospital of Chinese People’s Liberation Army, Beijing, 100853 China; 7 State Key Laboratory of Biocontrol, MOE Key Laboratory of Aquatic Product Safety, Institute of Aquatic Economic Animals and Guangdong Province Key Laboratory for Aquatic Economic Animals, School of Life Sciences, Sun Yat-sen University, Guangzhou, Guangdong, 510275 China; 8 Department of Biostatistics, University of Pittsburgh, Pittsburgh, PA 15261 USA; 9 Division of Pulmonary Medicine, Allergy and Immunology, Department of Pediatrics, Children’s Hospital of Pittsburgh of UPMC, Pittsburgh, PA 15224 USA; 10 Translational Health Sciences, University of Bristol, Bristol, UK; 11 National Institute for Health Research Biomedical Research Centre at Moorfields Eye Hospital NHS Foundation Trust and UCL Institute of Ophthalmology, London, UK

Correction to: *Cell Discovery* (2021) 7:13

10.1038/s41421-021-00245-6 published online 9 March 2021

In the original publication of this paper^1^, the accession number for the raw sequencing data is missing. The raw data have been deposited in the European Nucleotide Archive (ENA) in 2019 under the accession number PRJNA429692 that are publicly accessible at https://www.ebi.ac.uk/ena/browser/view/PRJNA429692. This correction does not affect the description of the results or the conclusion of this work.
