# Supplementary material for: Identification of an intraocular microbiota
Source: Cell Discov. 2021 Mar 9;7:13. doi: 10.1038/s41421-021-00245-6 (PMC7943566; doi:10.1038/s41421-021-00245-6)
Supplement: Supplementary file 1 — Supplementary Figures [file 41421_2021_245_MOESM1_ESM.pdf]

Supplementary Fig. S1

a

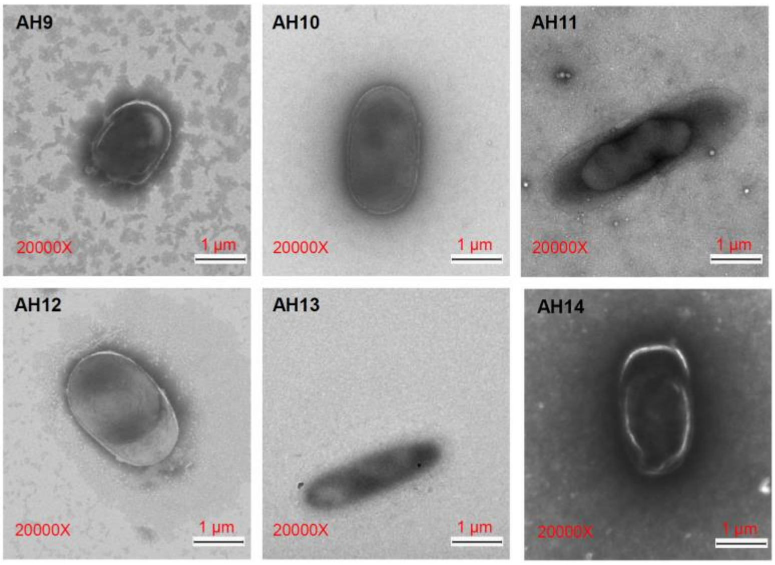

b

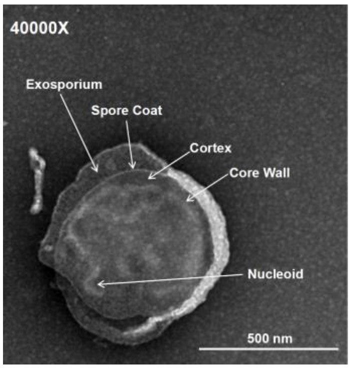

Supplementary Fig. S1. Negative staining transmission electron microscopy shows bacteria in minimally manipulated fresh additional AH specimens at 20,000X magnification (**a**) and free spores found in AH specimens at 40,000X magnification (**b**).

Supplementary Fig. S2

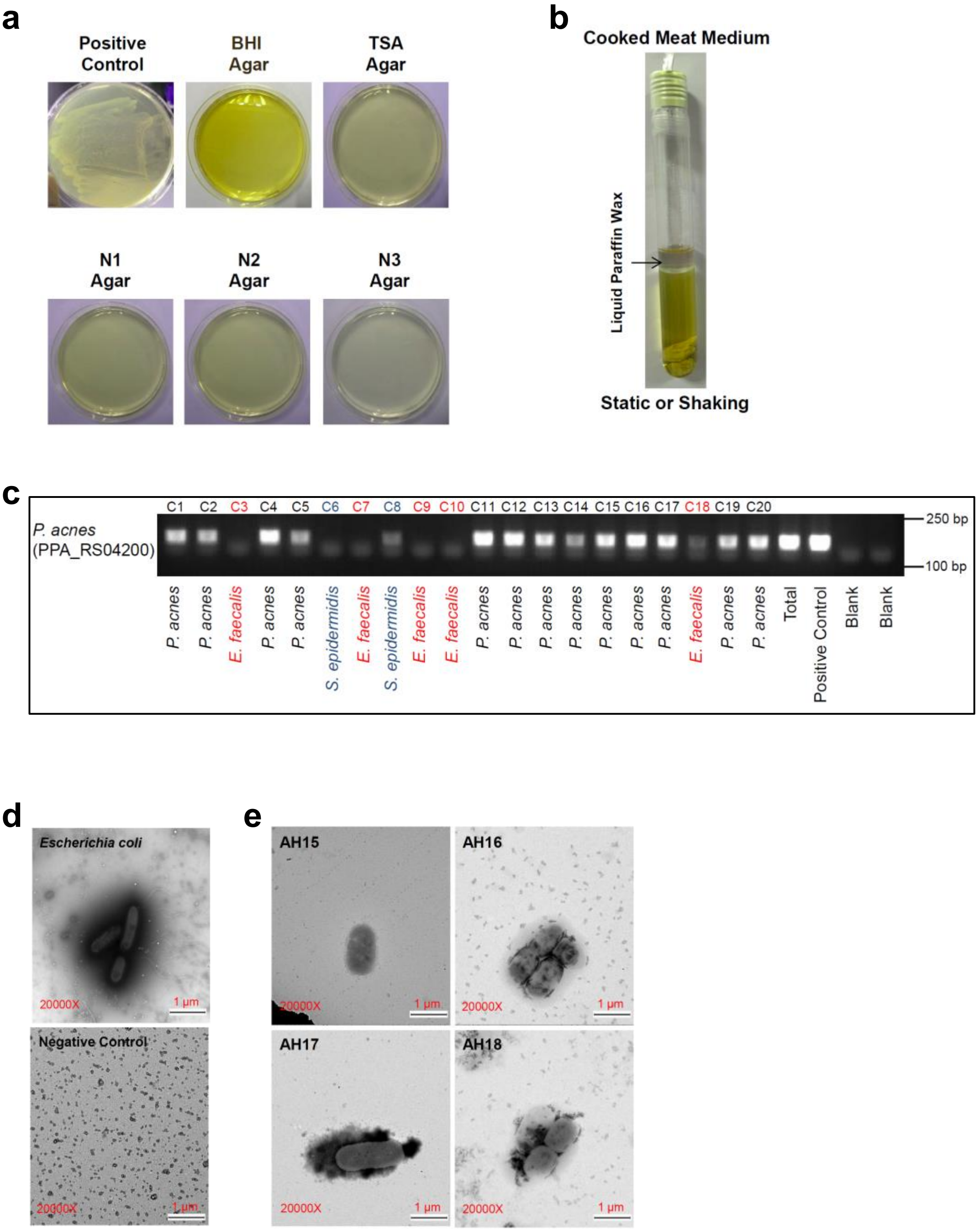

Supplementary Fig. S2. Culture of AH

(**a**) Cultures (negative) of AH in BHI, TSA, N1, N2, and N3 agar mediums. (**b**) Cultures in cooked meat medium covered by liquid paraffin wax. (**c**) Twenty randomly picked bacterial clones from anaerobic cultures of AH samples were subjected to high-throughput sequencing to clarify their identities. The PCR assays (with the primer pair of PPA_RS04200 detecting *P. acnes*) were used to confirm the identities of these 20 clones named C1-C20. Among all twenty clones, C3, C7, C9, C10, and C18 (in red) were

identified as *E .faecalis*, while C6 and C8 were identified as *S. epidermidis* (in blue). The cultured *P. acnes* was used as positive control. (**d**) Negative staining transmission electron microscopy visualized cultured *E. coli* and negative control (PBS without AH cultures) at 20,000X magnification. (**e**) Selected representative cultured AH samples at 20,000X magnification visualized by negative staining transmission electron microscopy.

Supplementary Fig. S3

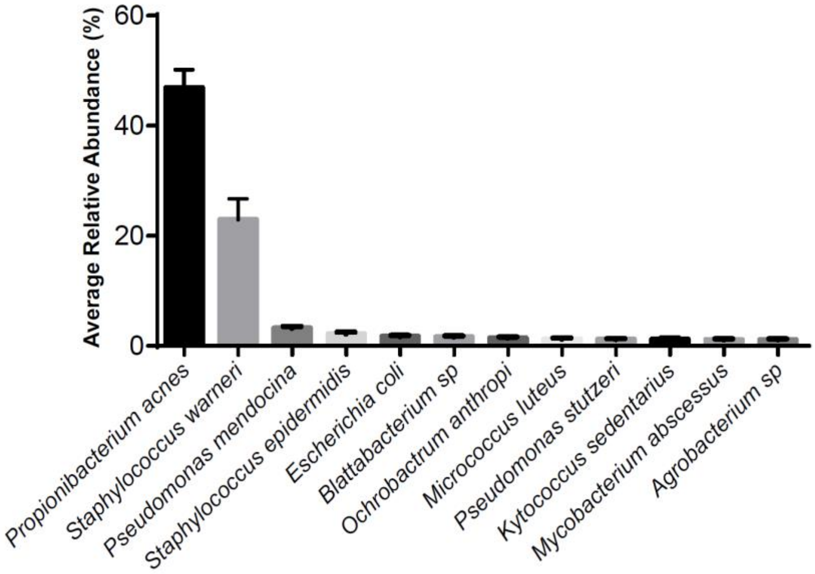

Supplementary Fig. S3. Twelve bacteria species found in AH samples with at least 1% of the average of relative abundance among all bacterial reads.

Supplementary Fig. S4

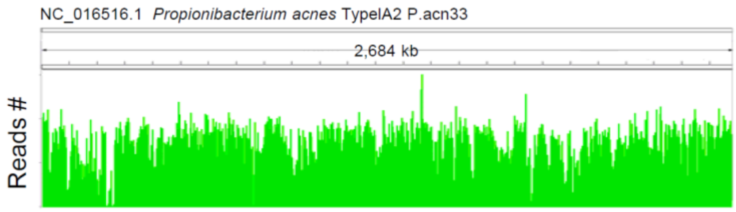

Supplementary Fig. S4. Alignment of all reads mapped to *P. acnes* to the template genome of *P. acn33*.

Supplementary Fig. S5

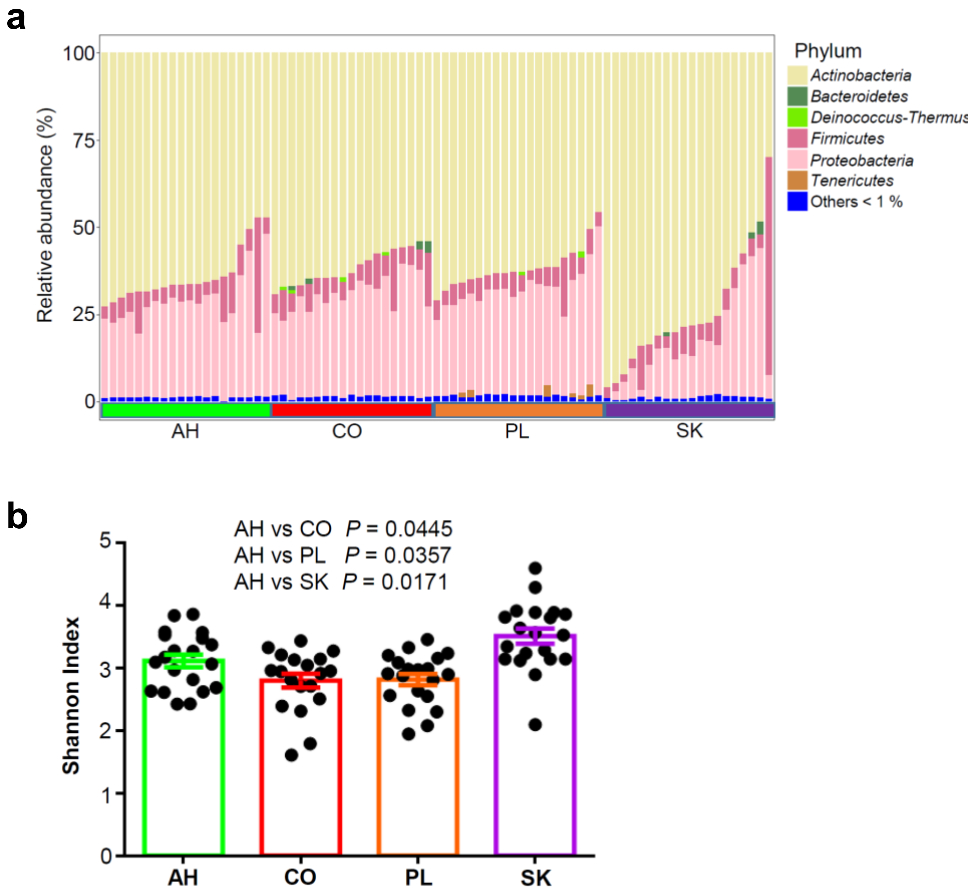

Supplementary Fig. S5. Relative abundance of the major phyla (**a**) of bacteria in the metagenomes of AH, CO, PL, and SK samples. The alpha diversity (measured by Shannon index) of the bacterial composition in the AH, CO, PL, and SK samples. *P* value was calculated using Mann-Whitney U test (**b**).

Supplementary Fig. S6

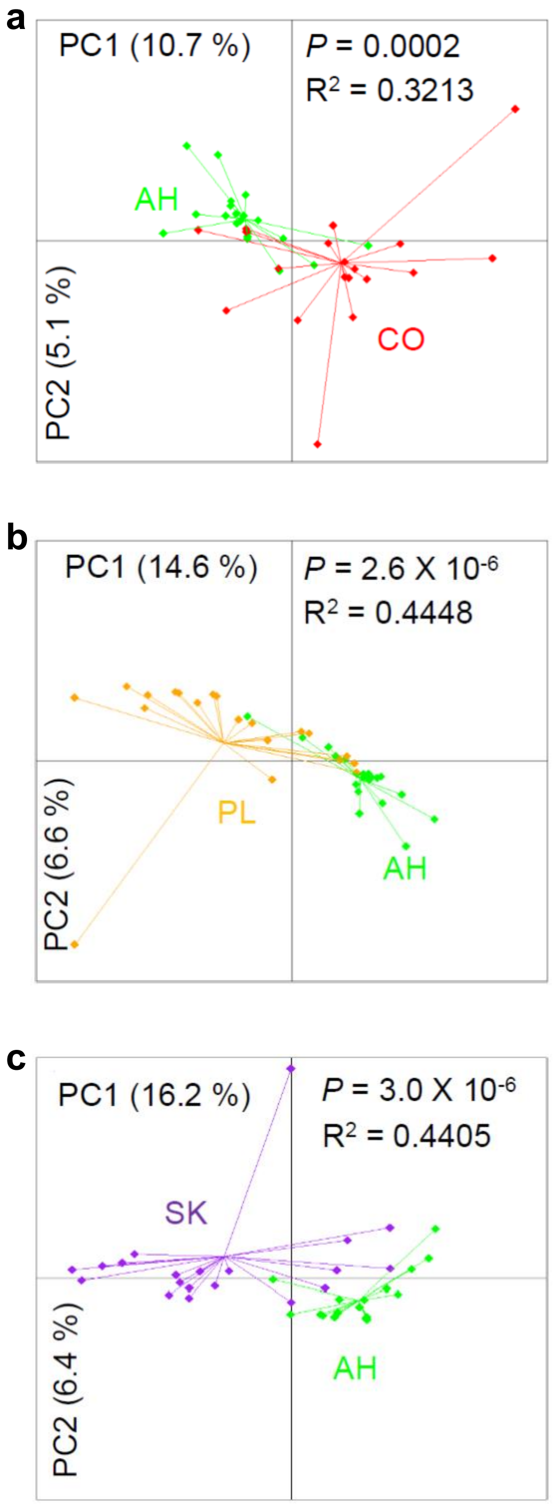

Supplementary Fig. S6. Similarity of the microbial community between (**a**) AH and Conjunctiva (CO), (**b**) AH and Plasma (PL), and (**c**) AH and Skin (SK) was analyzed by PCoA with Bray-Curtis distance. *P* value was calculated using PERMANOVA test.

Supplementary Fig. S7

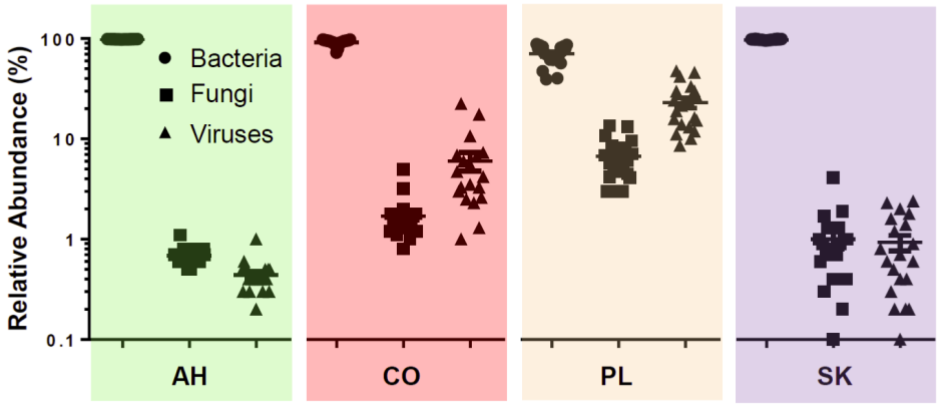

Supplementary Fig. S7. Relative abundance of bacteria, fungi, and viruses among AH, CO, PL, and SK samples analyzed by metagenomic sequencing.

Supplementary Fig. S8

a

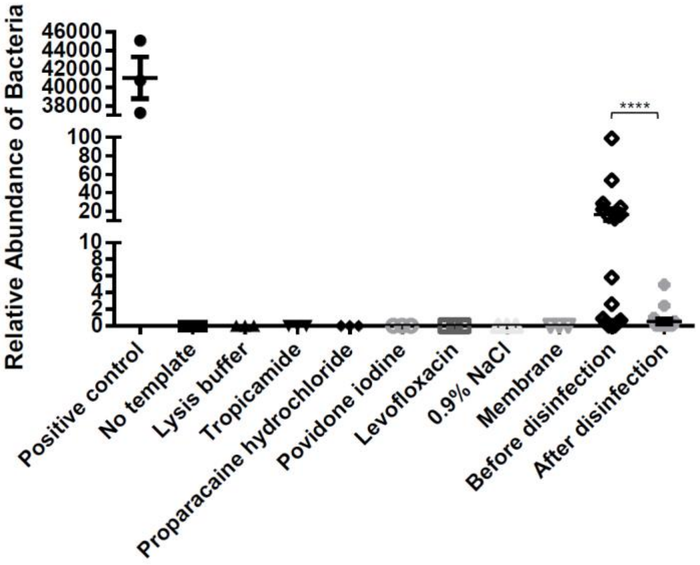

b

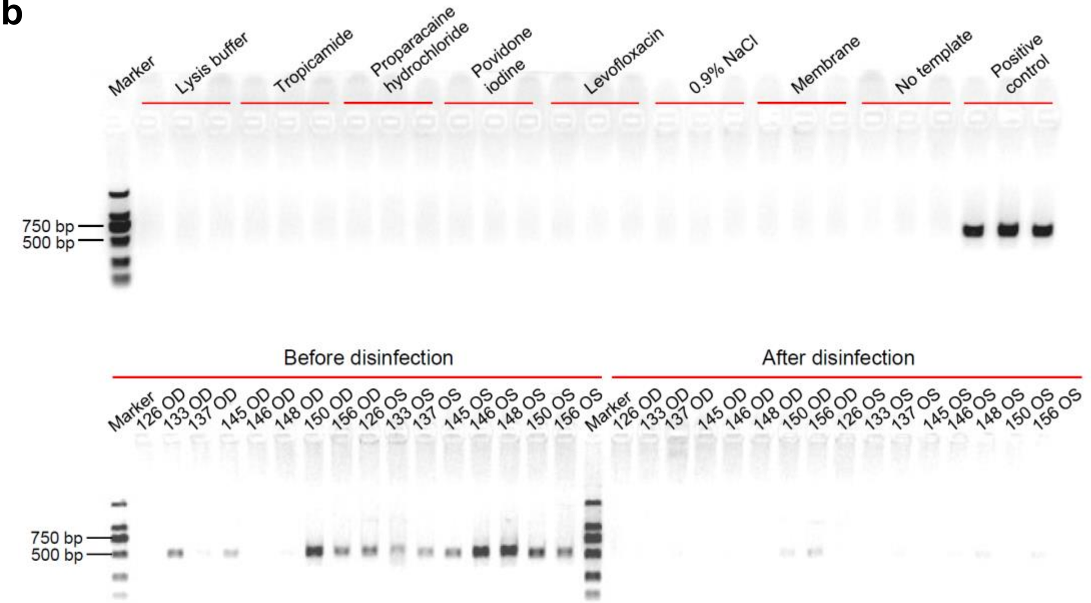

Supplementary Fig. S8. The relative abundance of bacteria on the conjunctiva of macaques before and after the disinfection process.

The abundance of bacteria relative to the conditions of “No template”, detected by 16S rDNA PCR assays, was measured in conjunctival samples obtained before and after the disinfection procedure mimicking the one performed on patients prior to cataract surgery. All reagents used in the disinfection procedure including tropicamide, proparacaine hydrochloride, povidone iodine, levofloxacin, 0.9% NaCl, as well as DNA extraction lysis buffer and the membrane used in conjunctival impression cytology sampling process were included as negative controls. Cultured P. acnes were used as the positive control for 16S rDNA PCR assays. The relative abundance of bacterial species was measured using

real-time PCR assays (**a**) and the PCR products after 40 cycles were visualized using Agarose gel electrophoresis (**b**). Mann-Whitney U test significance levels are denoted by asterisks (*****P*<0.0001).

Supplementary Fig. S9

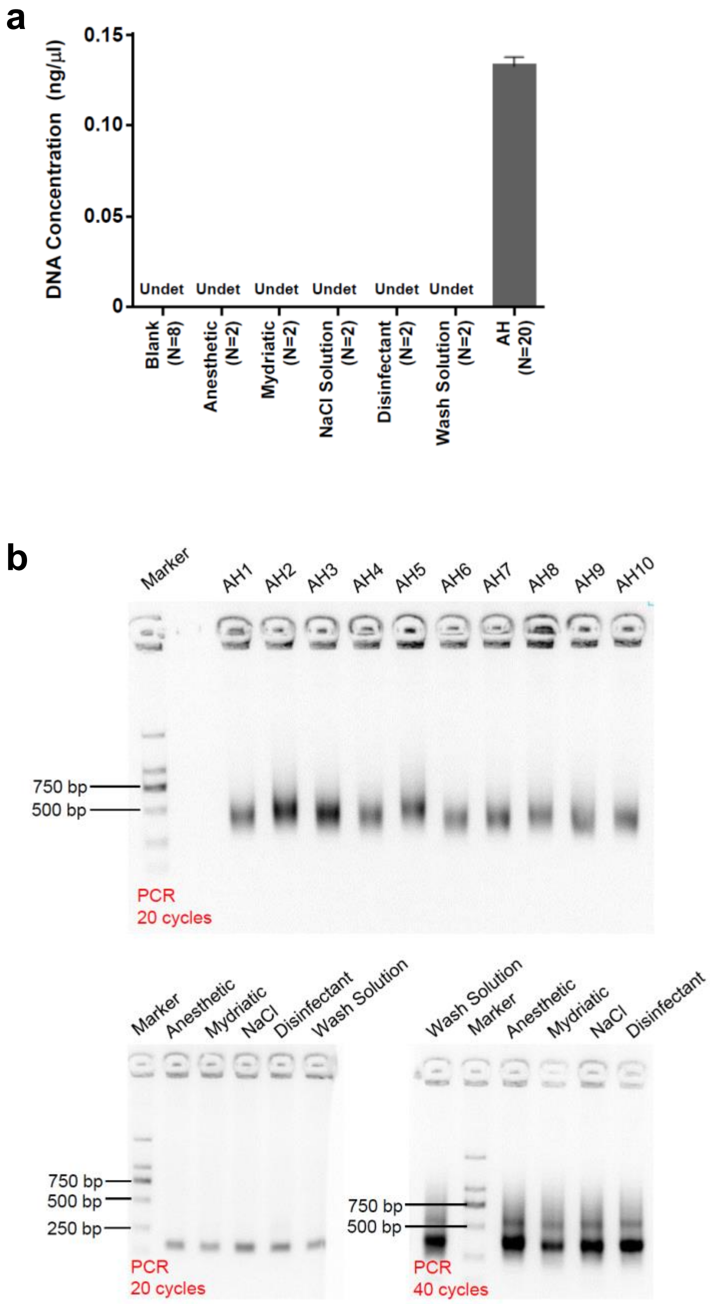

Supplementary Fig. S9. Controls for metagenomic sequencing experiments

(**a**) The average DNA concentration of Blank, Wash Solution, Anesthesia, Disinfectant, NaCl Solution, Mydriatic, and AH samples. (**b**) The DNA electrophoresis analysis of sequencing libraries of AH samples (upper panel), and Blank, Wash Solution, Anesthesia, Disinfectant, NaCl Solution, Mydriatic controls (lower panel).

Supplementary Fig. S10

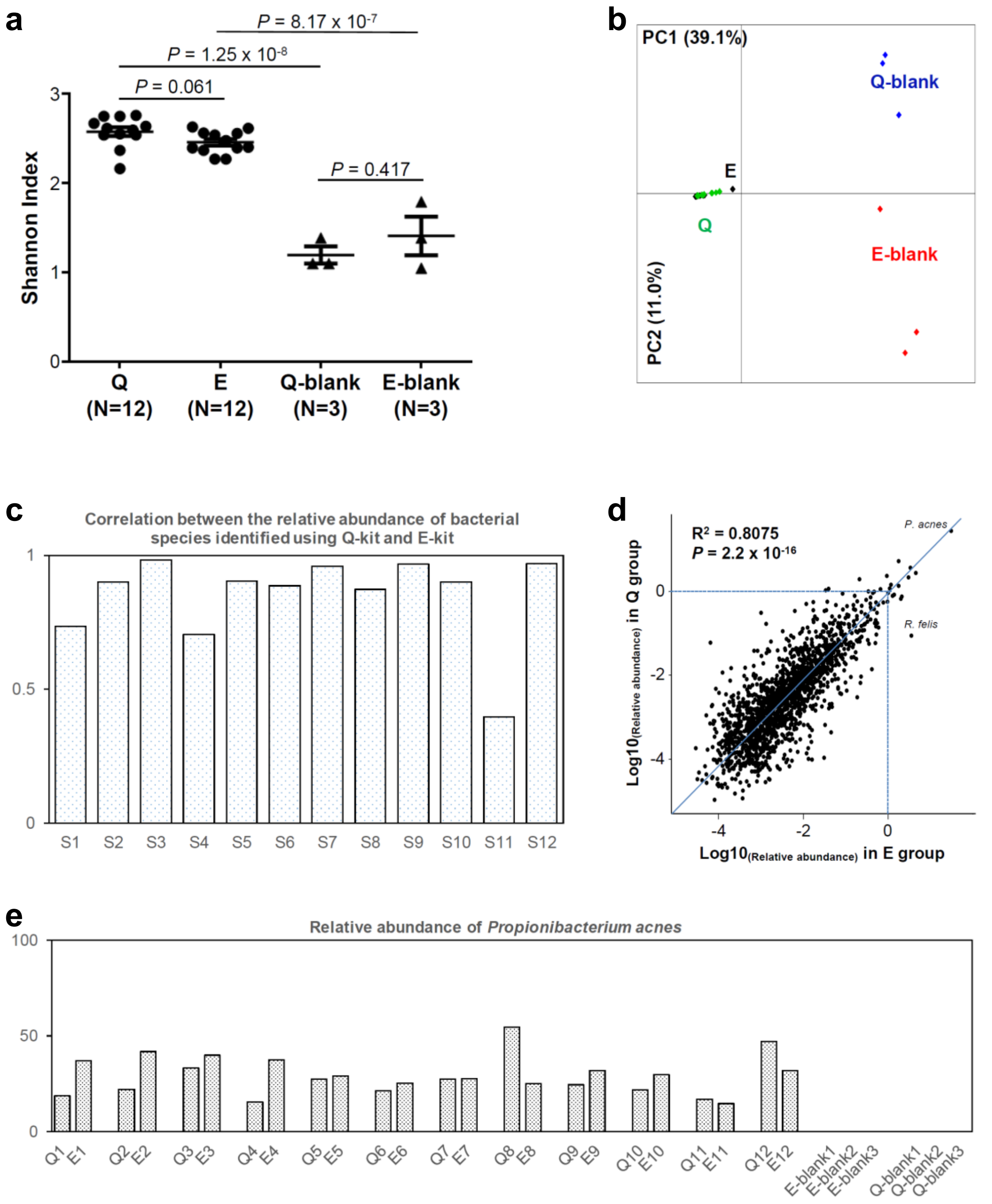

Supplementary Fig. S10. Bacterial communities identified using two DNA isolation kits (**a**) The alpha diversity (measured by Shannon index) of the intraocular bacterial communities in 12 patients with cataract, identified using either the Q kit (Q) or the E kit (E). Blank samples without any biological specimens served as negative controls for the Q (Q-blank, N=3) or E (E-blank, N=3) kits. The error bar represents the mean of all Shannon index within the group ± SEM. The statistical difference was measured between groups using Mann-Whitney U test. (**b**) PCoA of the similarity of the intraocular metagenomes. *P* value was calculated using PERMANOVA test. (**c**) The R value was calculated using Pearson correlation coefficient between paired bacterial communities identified using Q or

E kit. (**d**) The average relative abundance of each bacterial species in 12 communities identified using Q kit or 12 communities identified using E kit was compared in scatter plot. (**e**) The relative abundance of *P. acnes* in 12 communities identified using Q kit or 12 communities identified using E kit.

Supplementary Fig. S11

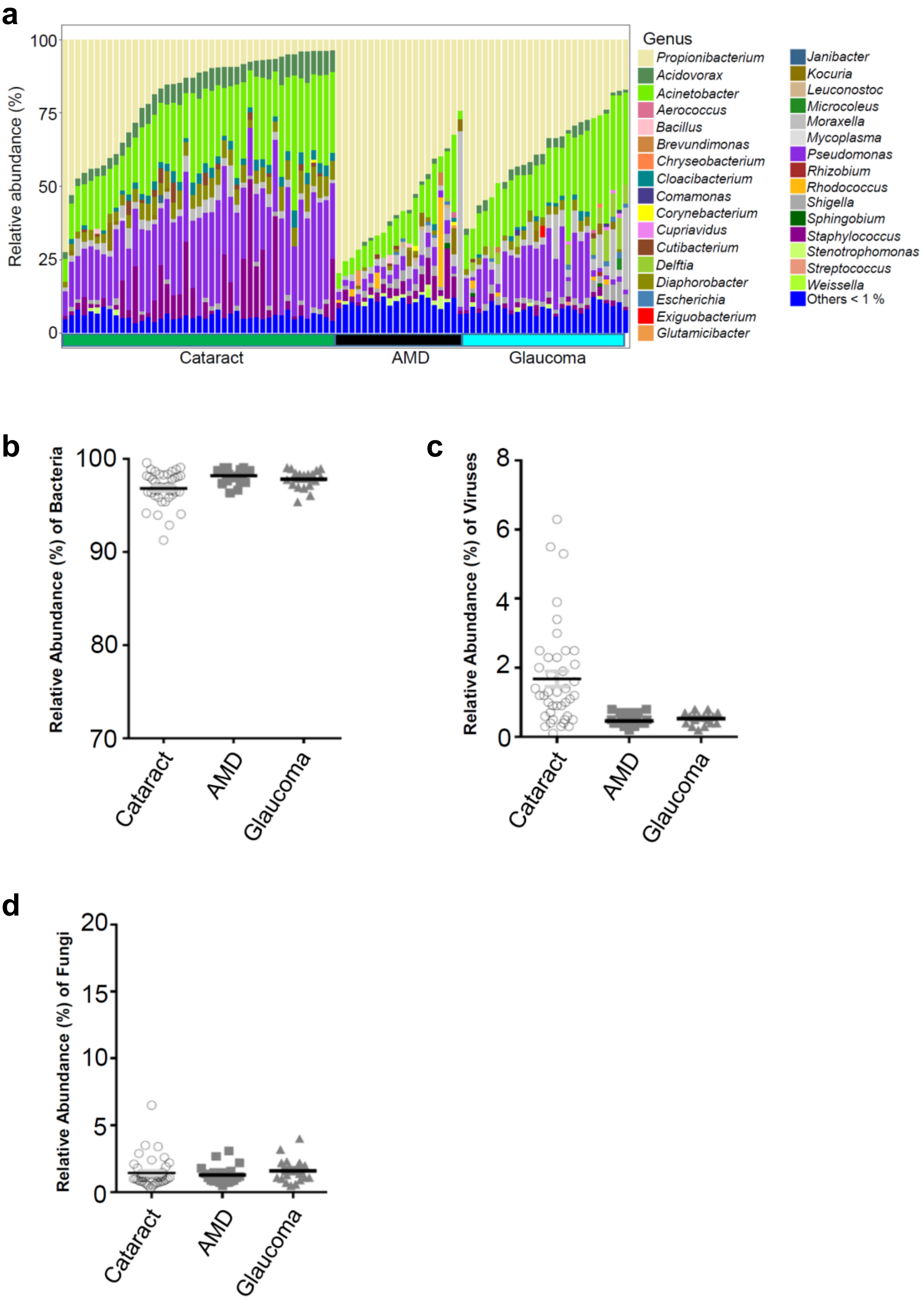

Supplementary Fig. S11. (**a**) Relative abundance of the major genera of bacteria in the intraocular metagenomes in cataract, AMD, and glaucoma patients. The relative abundance of bacterial (**b**), viral (**c**), and fungal (**d**) species of the intraocular metagenomes in patients with cataract, AMD, or glaucoma.

Supplementary Fig. S12

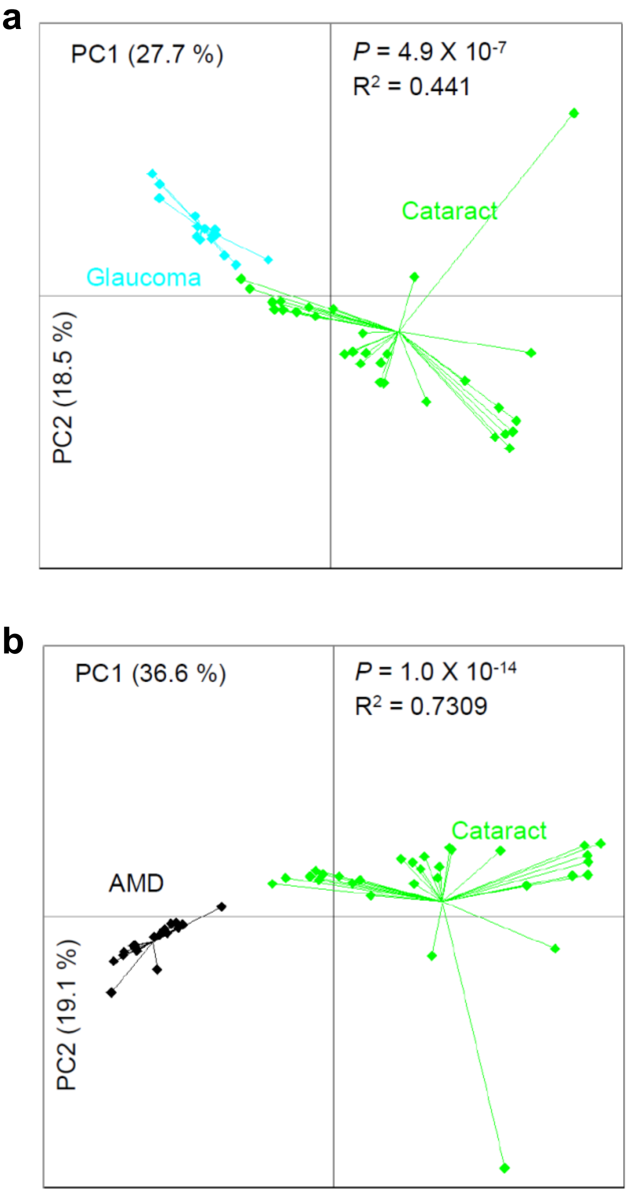

Supplementary Fig. S12. Similarity of the microbial community between cataract and glaucoma (**a**) or cataract and AMD (**b**) was analyzed by PCoA. *P* value was calculated using PERMANOVA test.

Supplementary Fig. S13

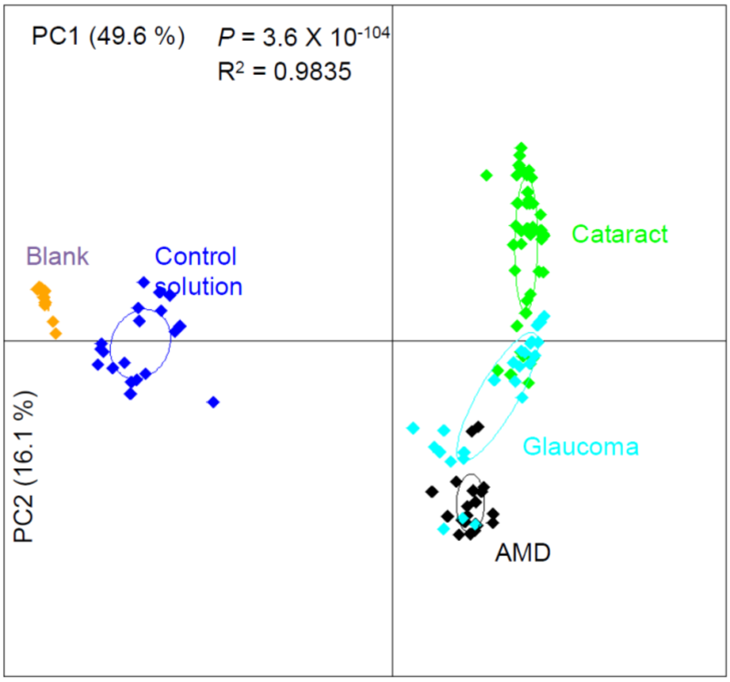

Supplementary Fig. S13. Similarity of the microbial community between patient specimens and controls was analysed by PCoA. Control solutions included Wash Solution, Anesthesia, Disinfectant, NaCl Solution, Mydriatic controls. *P* value was calculated using PERMANOVA test.

Supplementary Fig. S14

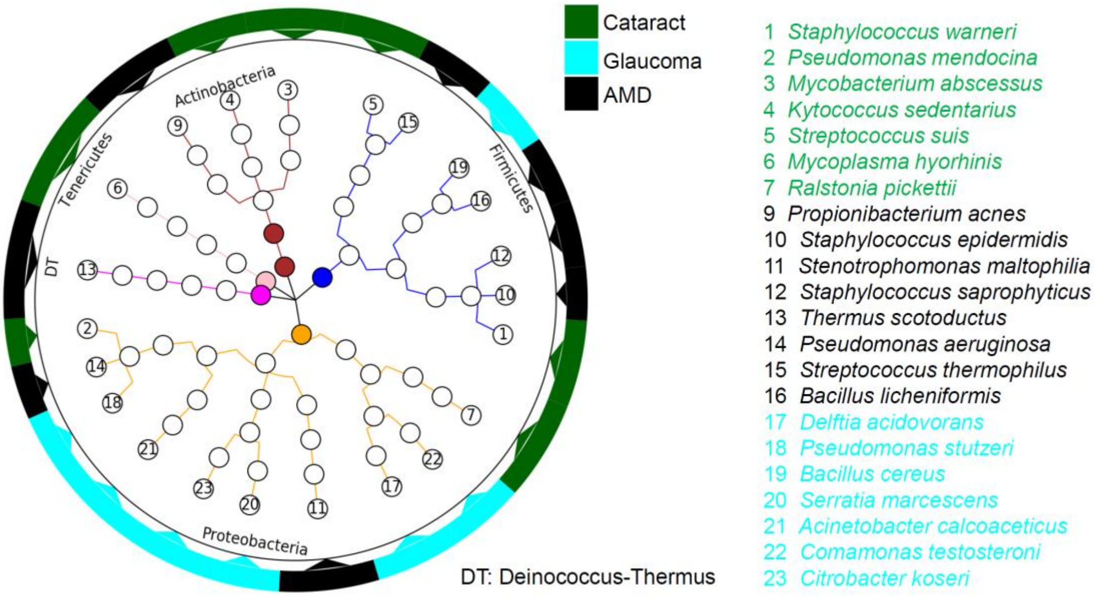

Supplementary Fig. S14. The bacterial species highly enriched in intraocular metagenomes in patients with cataract (in green), AMD (in black), and glaucoma (in blue), identified using LefSe, were clustered.

Supplementary Fig. S15

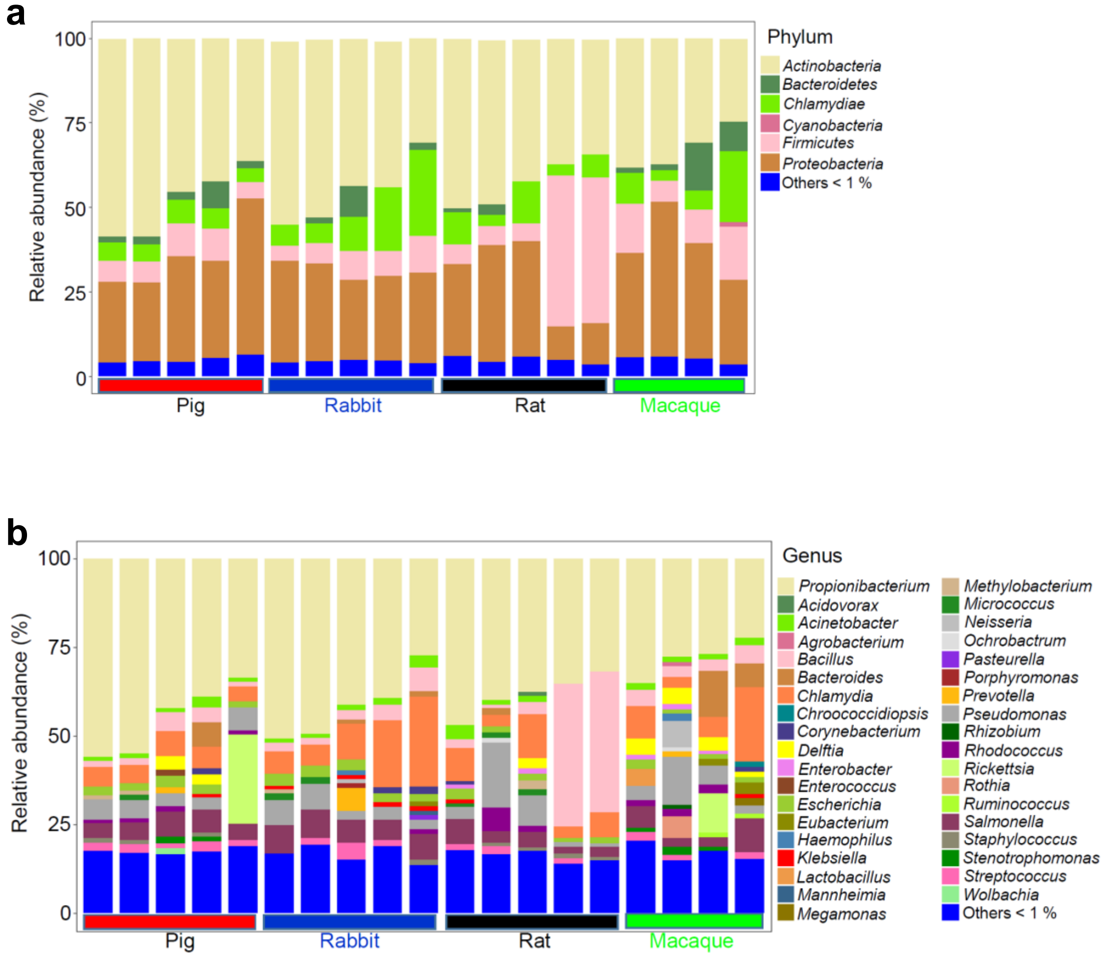

Supplementary Fig. S15. Relative abundance of the major phyla (**a**) and genera (**b**) of bacteria in the intraocular metagenomes in pig, rabbit, rat, and macaque.
