# Supplementary material for: Identification of an intraocular microbiota
Source: Cell Discov. 2021 Mar 9;7:13. doi: 10.1038/s41421-021-00245-6 (PMC7943566; doi:10.1038/s41421-021-00245-6)
Supplement: Supplementary file 2 — Supplementary Table S1 [file 41421_2021_245_MOESM2_ESM.pdf]

Supplementary Table S1. Demographic summary of study subjects and summary of direct metagenomic shotgun sequencing reads

	Cohort 1 Cataract	Cohort 2 Cataract	Cohort 3 Cataract				Cohort 4 Cataract	Cohort 5 AMD	Cohort 6 Glaucoma
	Aqueous Humor	Aqueous Humor	Aqueous Humor	Conjunctiva	Plasma	Skin	Aqueous Humor	Aqueous Humor	Aqueous Humor
# of Subject	1000	41	20	19	20	20	12	20	26
Ave Age	69.9	68.4	75.0	75.0	75.0	75.0	60.4	66.3	61.9
Male/Female	431/569	19/22	10/10	10/9	10/10	10/10	6/6	12/8	13/13
# of Sample	1000	41	20	19	20	20	24	20	26
Ave # of Total Reads	-	41,806,363	31,396,400	22,240,085	43,853,209	28,034,294	42,632,723	38,133,965	40,585, 200
Ave # of nonHuman Reads passed filter	-	15,903,458	1,681,502	1,961,170	1,579,702	2,374,707	2,420,576	13,074,569	13,699,000

Supplementary Table S2. Bacteria found in AH specimens from cataract patients (the relative abundance>0.1% in at least one sample)

Supplementary Table S3. Microbial metabolic pathways highly enriched in aqueous humor specimens

Supplementary Table S4. Bacteria identified in AH specimens using Q or E kits (relative abundance)

Supplementary Table S5. Bacteria found in AH specimens from cataract, AMD, and glaucoma patients (the relative abundance>0.1% in at least one sample)
